# Two to tango: endothelial cell TMEM16 scramblases drive coagulation and thrombosis

**DOI:** 10.1172/JCI170643

**Published:** 2023-06-01

**Authors:** János G. Filep

**Affiliations:** 1 Department of Pathology and Cell Biology, University of Montreal, Montreal, Quebec, Canada.; 2 Research Center, Maisonneuve-Rosemont Hospital, Montreal, Quebec, Canada.

## Abstract

Endothelial cells form a constitutively anticoagulant surface under homeostasis. While loss of this anticoagulant property is a hallmark of many cardiovascular diseases, the molecular mechanisms underlying the procoagulant transition remain incompletely understood. In this issue of the *JCI*, Schmaier et al. identify the phospholipid scramblases TMEM16E and TMEM16F, which support endothelial procoagulant activity through phosphatidylserine (PS) externalization. Genetic deletion of TMEM16E or TMEM16F or treatment with TMEM16 inhibitors prevented PS externalization and reduced fibrin formation in the vessel wall independently of platelets in a murine laser-injury model of thrombosis. These findings reveal a role for endothelial TMEM16E in thrombosis and identify TMEM16E as a potential therapeutic target for preventing thrombus formation.

## Phosphatidylserine modulation of hemostasis and thrombosis

Thrombosis is the most feared complication and a major pathomechanism of three major cardiovascular disorders, ischemic heart disease, stroke, and venous thromboembolism, which are leading causes of mortality among adults worldwide (1). The current concept of hemostasis proposes that vessel damage and exposure of subendothelial tissue factor (TF) generate trace amounts of thrombin, which has multiple effects on platelets and other coagulation factors. Activation of multiple amplifying loops in the coagulation system and platelet activation lead to the formation of large amounts of fibrin and stabilization of platelet thrombi (2). Activated platelets readily externalize anionic phospholipids, most commonly phosphatidylserine (PS), and shed PS-expressing microvesicles (3). PS binds to and allosterically regulates coagulation factors Xa and Va, promoting activation of factor X by the TF–factor VIIa complex and the factor Xa–factor Va–prothrombinase complex. These steps correspond with the initiating, amplification, and propagation phases in thrombin formation (3). Thus, PS exposure on activated platelets or platelet-derived microvesicles has been suggested as playing an integral role in the amplification and propagation of the coagulation process in both hemostasis and thrombotic diseases.

PS is an important constituent of plasma membrane phospholipids, but in resting cells is sequestered on the inner membrane leaflet (4). The lipid composition of either side of the cell membrane is controlled by active transporters (termed flippases and floppases) and passive scramblases (5). The TMEM16 family (also known as anoctamins) consists of 10 integral membrane proteins with diverse functions, including scramblases and channels (6). TMEM16A and B are Ca^2+^-activated chloride channels, whereas TMEM16C, -D, -F, -G, and -J function as Ca^2+^-activated phospholipid scramblases and/or nonselective ion channels (7). Recessive mutations in *TMEM16E* are associated with two different muscular dystrophies, proximal limb-girdle muscular dystrophy and distal Miyoshi myopathy, possibly due to loss of a Cl^–^ current needed during membrane repair in muscle (7, 8). Truncations and missense variants of *TMEM16K* and *TMEM16C* cause the autosomal recessive spinocerebellar ataxia SCAR10 and craniocervical dystonia, respectively (7). There are no reports on whether any of these conditions are associated with abnormal hemostasis or thrombosis. Mutations in *TMEM16F* underlie Scott syndrome, characterized by defective exposure of PS on the outer membrane leaflet of platelets upon Ca^2+^-dependent activation and decreased levels of coagulation factors (9, 10). In contrast, PS externalization induced by a proapoptotic stimulus was only partially inhibited in the platelets from these patients, suggesting the existence of at least two distinct mechanisms of phospholipid scrambling (11). Platelet- and megakaryocyte-specific loss of TMEM16F in mice resulted in a mild bleeding disorder resembling that of patients with Scott syndrome (12, 13). However, in spite of substantial defects in hemostasis and reduction in arterial thrombus formation, TMEM16F deficiency did not ameliorate ischemic stroke (14). These findings highlight differences between the roles of platelets and coagulation in thrombosis, but cannot explain the complex regulation of PS externalization beyond TMEM16F. Moreover, conventional antiplatelet therapies cannot completely prevent thrombotic events, implying a therapeutic gap due to other yet unappreciated mechanisms.

## TMEM16E and TMEM16F promote endothelial cell procoagulant activity

**Figure 1:**
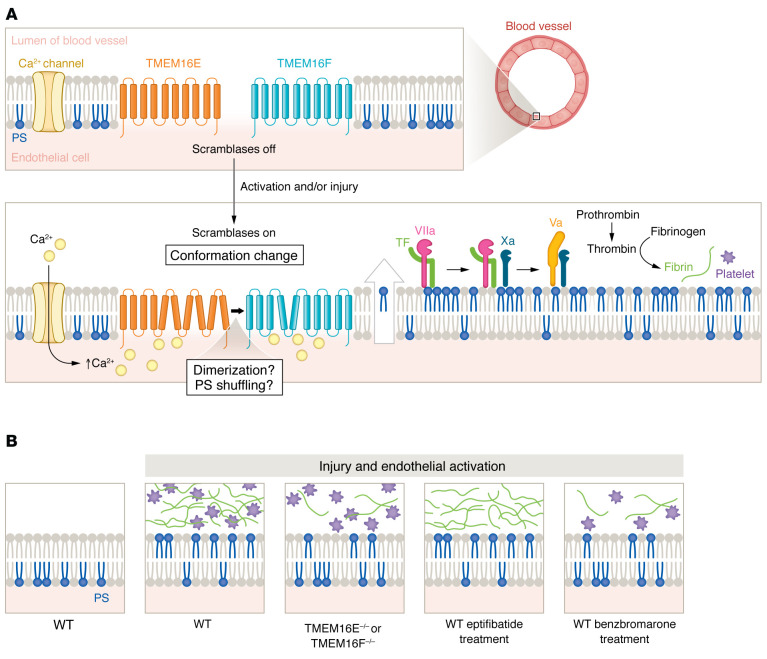
TMEM16E and TMEM16F regulate endothelial cell procoagulant activity and thrombosis. (**A**) Endothelial cells express the Ca^2+^-activated phospholipid scramblases TMEM16E and TMEM16F. Activation and/or injury triggers increases in intracellular Ca^2+^ and conformation changes in scramblases, leading to externalization of anionic phospholipids, most commonly PS. TMEM16E and TMEM16F might be epistatic to one another or function in a linear pathway. One possible mechanism involves the shuttling of PS to the plasma membrane by TMEM16E, where it can be directly externalized by TMEM16F. Alternatively, TMEM16E and TMEM16F could form a heterodimer in which TMEM16E functions as a regulator of TMEM16F. The precise localization of TMEM16E and TMEM16F, within the same or separate compartments, remains unclear. Externalized PS binds to and allosterically regulates coagulation factors Xa and Va, promoting activation of factor X by the TF–factor VIIa complex and the factor Xa–factor Va–prothrombinase complex, leading to thrombin formation and fibrin generation. (**B**) Laser injury to the vessel wall induces PS externalization on the endothelium, fibrin deposition, and platelet accumulation at the injured sites in mice. Mice with genetic depletion of either TMEM16E or TMEM16F showed reduced PS externalization and fibrin deposition, but platelet accumulation similar to that of WT littermates. Prevention of platelet accumulation with eptifibatide did not reduce PS externalization on the endothelium or diminish fibrin formation. Treatment with the TMEM16 inhibitor benzbromarone reduced platelet accumulation, endothelial PS externalization, and fibrin deposition. These results indicate that PS externalization on the vessel wall drives thrombosis.

In this issue of the *JCI*, Schmaier and coauthors identified five proteins as important regulators of factor VIIa–catalyzed activation of factor X in TNF-α–stimulated human umbilical vein endothelial cells (HUVECs), including TMEM16F, TMEM16E, the closest paralog of the canonical Ca^2+^-activated scramblase TMEM16F (6), and Xkr9, a member of the Xk-related family of caspase-activated phospholipid scramblases (15). Expression of TMEM16E and TMEM16F was confirmed in unstimulated HUVECs and primary endothelial cells from human coronary artery and microvascular tissue, whereas *XKR9* expression was undetectable with quantitative PCR (qPCR) in these cell types. Silencing of either TMEM16E or TMEM16F resulted in a similar (approximately 50%) reduction of factor Xa generation and factor VIIa–catalyzed factor X activation. The degree of inhibition was similar to that observed following treatment with lactadherin, which neutralizes externalized PS. HUVECs stimulated with TNF-α, the Ca^2+^ ionophore A23187, or lipopolysaccharide also required TMEM16E and TMEM16F to support thrombin generation in human plasma. Consistently, silencing of TMEM16E or TMEM16F markedly reduced PS externalization in HUVECs in response to TNF-α or A23187 without affecting intracellular Ca^2+^ flux, TF cell-surface expression, or TF pathway inhibitor (TFPI) expression (16). Furthermore, pharmacological blockade of TMEM16 proteins with the unrelated small molecules benzbromarone and CaCCinh-A01 inhibited Ca^2+^-ionophore–stimulated PS externalization in parallel with activation of factor X, but without affecting intracellular Ca^2+^ flux (15). These results indicate that TMEM16E and TMEM16F regulate endothelial procoagulant activity through PS externalization (Figure 1A).

An intriguing finding of Schmaier et al. is the absence of additive suppression for factor Xa generation following dual silencing of TMEM16E and TMEM16F (15). Thus, TMEM16E and TMEM16F appear to be epistatic to one another or to function in a linear pathway. A possible mechanism for independent activities could involve the step-wise shuttling of PS, first by TMEM16E to the plasma membrane, then by TMEM16F to externalize it from the plasma membrane. Alternatively, TMEM16E could function as a regulator of TMEM16F, perhaps by forming a heterodimer, as TMEM16 proteins are capable of assembling into homo- and heterodimers (7, 17). Whether TMEM16E and TMEM16F interact in so-to-speak tango or shuffle or whether they are located in the same or separate compartments remains to be investigated. Of note, a recent study implicates TMEM16E as having a role in coordinating membrane fusion between muscle precursor cells to produce multinucleated skeletal muscle fibers (18).

## PS externalization on the vessel wall drives thrombosis

Schmaier and coauthors used intravital microscopy to monitor PS externalization and platelet and fibrin accumulation in cremaster arterioles following laser injury to the vessel wall and observed PS externalization along the injured vessel wall, not in the growing platelet aggregate (15). PS externalization also spread to the opposite wall in about one-third of the injuries without platelet accumulation or fibrin formation, indicating that these processes require adhesion proteins or TF in addition to PS externalization. Unexpectedly, prevention of platelet accumulation at the site of injury with the glycoprotein IIb/IIIa antagonist eptifibatide did not affect PS externalization. These observations were corroborated in TMEM16E-deficient (TMEM16E^–/–^) mice, which showed platelet accumulation similar to that of WT littermates, but diminished PS externalization and fibrin deposition, even in the absence of eptifibatide following laser injury. However, TMEM16E^–/–^ mice had no excessive bleeding (assessed in a tail-clip–bleeding assay), indicating that TMEM16E is not required for hemostasis. Pretreatment of WT mice with benzbromarone reduced platelet accumulation, PS externalization, and fibrin formation following laser ablation (Figure 1B). The beneficial effects of benzbromarone were still detectable when platelet accumulation was blocked with eptifibatide (15). Another study reported that prevention of PS externalization on platelets by platelet-specific deletion of phosphatidylinositol transfer protein-α (PITPα) did not prevent thrombus formation in a ferric chloride model (19).

## Translational implications and future directions

Important observations in Schmaier et al. (15) challenge the platelet-centered view of thrombus formation by phospholipid regulation and offer insights into how phospholipids externalized on the vessel wall can support thrombosis. The findings unveil a function of TMEM16E (in cooperation with TMEM16F) in the control PS externalization within endothelial cells, endowing the endothelium with procoagulant activity (15). Nevertheless, some limitations and potential areas of expansion are worthy of consideration.

While the authors’ data infer codependence of TMEM16E and TMEM16F in PS externalization, the underlying mechanism remains to be investigated (15). Scramblases may function in a cell-type–specific manner, as exemplified by the contribution of TMEM16F to procoagulant activity in platelets (11, 12), which do not express TMEM16E (15). Future studies might focus on compartmentalization of TMEM16 proteins and regulatory mechanisms of dimerization. Likewise, investigations into TMEM16 regulation of microvesicle release from endothelial cells will address another important aspect of thrombus propagation. Further studies should also address signaling pathways within endothelial cells to segregate pathways that are essential for triggering thrombus formation and those that mediate nonhemostatic functions (e.g., PS-dependent procoagulant activity compared with removal of apoptotic endothelial cells).

The drugs used in Schmaier et al. might have exerted additional effects on in vivo thrombus formation independently of inhibition of TMEM16 proteins, given the multiple pharmacologic targets of benzbromarone and CaCCinh-A01, including the inhibition of cytochrome P450 pathways and ROS production by xanthine oxidase, which can contribute to thrombus formation and propagation. Although benzbromarone did not affect standard coagulation tests in mice (15) and there are no reports of bleeding as an adverse effect in patients, reports on hepatotoxicity raise concerns about its clinical use. Results from a recent study on the structural basis for the activation of TMEM16F will likely aid the development of inhibitors (20). From a translational perspective, such data are critical for improving prevention or therapeutic targeting of thrombus formation.

In conclusion, this exciting study demonstrates that an activated endothelial surface, through expression of procoagulant phospholipids, contributes to thrombus formation and points toward TMEM16 scramblases as potential therapeutic targets for the prevention of the deleterious consequences of thrombus formation in cardiovascular diseases.

## Version 1 06/01/2023

Electronic publication
